# Bile acid profile associated with CSF and PET biomarkers in Alzheimer’s disease

**DOI:** 10.1007/s40520-024-02729-3

**Published:** 2024-03-07

**Authors:** Fardin Nabizadeh, Parya Valizadeh, Mohammad Sadegh Fallahi, Alzheimer’s disease Neuroimaging Initiative

**Affiliations:** 1 School of Medicine, Iran University of Medical Sciences, Tehran, Iran; 2 School of Medicine, Tehran University of Medical Science, Tehran, Iran

**Keywords:** Gut-brain axis, Alzheimer’s disease, Amyloid βeta, Bile acid, Tau, Metabolomics

## Abstract

**Background:**

Recent studies have shown that gut microbiota can affect the development of Alzheimer’s disease (AD) through various mechanisms. Bile acids (BAs), which are the final byproducts of cholesterol metabolism created through both the human body and gut microbiome, appear to be influenced by gut microbiota and may impact AD pathological characteristics such as the accumulation of tau and amyloid-β. We aimed to investigate the associations between various serum BAs and CSF biomarkers (including Aβ, total tau, and p-tau). Additionally, we sought to examine the longitudinal changes in brain Aβ and tau through PET imaging in relation to BAs profile.

**Methods:**

The data of 828 subjects including 491 diagnosed with mild cognitive impairment (MCI), 119 patients diagnosed with AD, and 267 cognitively normal (CN) participants were obtained from ADNI. The baseline and longitudinal [^18^F] florbetapir and [^18^F] flortaucipir PET standard uptake value ratios (SUVR) measures were obtained to assess the accumulation of tau and Aβ. Moreover, baseline levels of serum BAs and CSF Aβ1–42, tau, and p-tau were used.

**Results:**

After FDR correction we observed that five BAs level and relevant calculated ratios were associated with CSF p-tau and tau, three with CSF Aβ1–42. Furthermore, three BAs level and relevant calculated ratios were associated with the tau-PET rate of change, and two with the Aβ rate of change.

**Conclusion:**

The findings from our study suggest a correlation between altered profiles of BAs and CSF and imaging biomarkers associated with AD. These results provide supporting evidence for the link between the gut microbiome and the pathological features of AD.

## Introduction

The gut-brain axis is a bidirectional communication system between the gastrointestinal tract and the central nervous system, which plays a crucial role in maintaining overall health. Recent studies have shown that the gut microbiota can affect the development of Alzheimer’s disease (AD) through various mechanisms, including neuroinflammation, amyloid-beta (Aβ) deposition, and tau protein accumulation. The gut microbiota can affect AD through several pathways, including the modulation of neurochemical and neurometabolic pathways, synthesis and secretion of neurotrophic factors, and the production of microbial molecules that influence brain function [1].

Bile acids (BAs), essential for the digestion and absorption of dietary fats, are synthesized from cholesterol in the liver. This process involves various enzymatic reactions, leading to modifications of the cholesterol molecule and the formation of bile acids with detergent-like properties. BA synthesis occurs through two major pathways: the neutral pathway, which modifies the steroid ring before side-chain cleavage, and the acidic pathway, which follows the opposite order. Enzymes located in different cellular compartments catalyze these reactions. Cholesterol 7α-hydroxylase initiates the neutral pathway and produces primary BAs, cholic acid, and chenodeoxycholic acid. Sterol 27-hydroxylase contributes to BA synthesis through the acidic pathway, particularly in specific conditions [2]. Following synthesis, most BAs are conjugated to glycine or taurine to enhance solubility and ionization. Conjugation prevents precipitation, minimizes absorption, and protects BAs from enzymatic cleavage. In the intestine, conjugated BAs are deconjugated and metabolized by gut bacteria, forming secondary BAs such as deoxycholic acid (DCA) and lithocholic acid (LCA) [3]. The enterohepatic circulation ensures the efficient recycling of BAs. BAs are secreted into bile, stored in the gallbladder, and released into the intestine during digestion. The majority of BAs are reabsorbed in the terminal ileum and transported back to the liver, maintaining a constant BA pool. BAs lost in feces are replenished by de novo synthesis in the liver [4].

BAs have been receiving growing attention in the context of AD and its potential link to the brain-gut-microbiota (BGM) axis [5]. BAs have the potential to regulate various aspects of the BGM axis, encompassing neural, immune, and neuroendocrine pathways. Within these pathways, secondary BAs, which are produced by microorganisms, can influence each of these processes. The pathologic effects of BAs may occur through multiple mechanisms, such as activating the farnesoid X receptor and inhibiting BA synthesis in the brain, blocking NMDA receptors, reducing levels of brain oxysterols, and interfering with the actions of 17β-estradiol [6].

To further address these controversies and overcome the limitations of the previous studies including small sample size and cross-sectional design, we aimed to investigate the associations between various serum BAs and cerebrospinal fluid (CSF) biomarkers (including Aβ, total tau, and p-tau). Additionally, we sought to examine the longitudinal changes in brain Aβ and tau through PET imaging in relation to BAs profile. By doing so, we aimed to provide a more comprehensive understanding of the associations between serum BAs and AD biomarkers while considering the dynamic changes over time.

## Materials and methods

### Data acquisition

The Alzheimer’s Disease Neuroimaging Initiative (ADNI) database provided the data for this investigation (adni.loni.usc.edu). The ADNI was founded in 2003 as a public-private partnership directed by Principal Investigator Michael W. Weiner, MD. The initial purpose of ADNI is to assess the progression of mild cogntive impairment (MCI) and early AD by combining all serial PET, MRI, biological markers, and clinical and neuropsychological measures. www.adni-info.org has the most up-to-date information.

### Participants

In this study, we utilized the data collected from the baseline visits of participants in the ADNI cohort who had available serum BA levels, CSF biomarkers, and longitudinal PET. From the dataset, we included participants aged between 55 and 90 years, who had a minimum of 6 years education, fluent in either Spanish or English, without any significant neurological disorder other than AD, and had available baseline serum BA levels, baseline CSF Aβ1–42, tau, and p-tau, available longitudinal Aβ-PET and tau-PET scans. Specifically, we included 491 individuals diagnosed with MCI, 119 patients diagnosed with AD, and 267 cognitively normal (CN) participants. All individuals with MCI were identified as having amnestic MCI based on specific criteria, which included a memory complaint, an education-adjusted score on the Wechsler Memory Scale Logical Memory II test indicating objective memory loss, a Mini-Mental State Examination (MMSE) score between 24 and 30, a Clinical Dementia Rating (CDR) of 0.5, preserved activities of daily living, and no significant impairment in other cognitive domains or signs of dementia. The ADNI participants with AD were diagnosed according to the National Institute of Neurological and Communicative Disorders and Stroke–Alzheimer’s Disease and Related Disorders Association (NINCDS-ADRDA) probable AD criteria, with MMSE scores ranging from 20 to 26 and CDR of either 0.5 or 1.

### Assessment of CSF Aβ1–42, tau, and p-tau

The Luminex platform was used to obtain CSF samples, and the levels of specific CSF biomarkers, including Aβ1–42, total tau, and p-tau, were measured using Luminex’s micro-bead-based multiplex immunoassay. Additional information regarding the collection of CSF specimens and analytical measurement can be found on the ADNI website (http://adni.loni.usc.edu/methods/documents/).

### PET imaging biomarkers

Aβ deposition was visualized with PET tracer [^18^F]AV45 [7]. The measurements of regional Aβ standard uptake value ratios (SUVR) that were calculated by the ADNI core at UC Berkeley, which they accessed through adni.loni.usc.edu were acquired. To determine global Aβ deposition, we standardized the mean uptake in several regions of interest (ROIs), including prefrontal, orbitofrontal, parietal, temporal, anterior cingulate, and posterior cingulate/precuneus, by dividing it by the cerebellum and composite reference region for cross-sectional and longitudinal analysis, respectively. We obtained Aβ SUVR values at various time points to estimate the rate of change.

Data of tau accumulation measured by tau-PET were retrieved from the ADNI server (Jagust Lab, UC Berkeley). The image processing techniques were previously described [8]. Tau-PET SUVR was defined by averaging flortaucipir (AV-1451) uptake in the Braak stage composite regions divided by a reference region such as inferior cerebellar GM (cross-sectional) or hemispheric WM (Longitudinal). The rate of change of tau-PET was estimated using available data of baseline and follow-up visits.

### Serum bile acids profile

We used quantitated data of BAs in human serum samples provided by Duke University for the ADNI GO/2 and ADNI 1 projects. The samples were stored at -80 °C until preparation and analysis using a bile acid-free matrix (BAFM) prepared using a charcoal-stripping protocol to minimize analytical variations and compensate for matrix effects. Sample preparation involved spiking each 50 µL of serum with 150 µL of acetonitrile containing internal standards, followed by extraction of bile acids using a laboratory shaker and centrifugation. The supernatant was lyophilized, reconstituted, and analyzed using ultra-performance liquid chromatography coupled with tandem mass spectrometry (UPLC-MS/MS). Quality control (QC) samples, including test mixtures and pooled biological samples, were used along with internal standards to monitor the stability of the large-scale analysis. Any metabolite with signal-to-noise ratio < 3.0 was rejected from statistical analysis, and the RSDs for each metabolite in the test mixtures and pooled QC samples measured were calculated. The study found that the RSDs of the lower concentration metabolites in the reference standard mixture were less than 30%, and the R.S.D. for the higher concentration metabolites shall be better than 15% for each batch of sample analysis. Overall, the study provides a reliable method for quantifying bile acids in human serum samples, which could facilitate the diagnosis and treatment of diseases associated with altered bile acid metabolism. The level of 33 serum BAs and 3 ratios were entered into the analysis.

The CA: chenodeoxycholic acid (CDCA) ratio was picked to investigate whether a change in the production of BAs occurs in the liver, moving from the primary to the alternative pathway. Additionally, the DCA:CA and glycolithocholic acid (GLCA):CDCA ratios were analyzed to observe variations in the gut microbiome’s enzymatic activity that might result in differences in the creation of secondary BAs.

### ApoE genotyping

The APOE genotyping of MCI patients was performed on collected blood samples. The participants with at least one ε4 allele are considered carriers, as described by ADNI (http://adni.loni.usc.edu/methods/documents/).

### Cognitive assessments

All participants underwent Mini-Mental State Examination (MMSE) which included 30 questions to measure the cognitive status at baseline. The MMSE score of patients was downloaded from ADNI.

### Statistical analysis

The difference in demographical, clinical, and CSF biomarkers between groups was measured using ANOVA analysis. To investigate the association between serum Bas level and CSF and PET biomarkers, multivariable linear regression models adjusted for the effect of age, sex, APOE ε4, and BMI were used. To address the false discovery rate (FDR) due to the multiple comparisons, we used the Benjamini-Hochberg method. R software (version 3.3.3; R Foundation for Statistical Computing, Vienna, Austria) was used for statistical analysis.

## Results

### Participant’s characteristics

The demographical and clinical characteristics of the participants are detailed in Table 1. The level of 33 serum BA levels and 3 ratios were available for analysis (Table 2). The average time interval between the baseline and follow-up scans for Aβ-PET and tau-PET were 2.12 (± 1.88) and 1.65 (± 1.72) years respectively.

**Table 1:** Participants characteristics

Characteristic	CN (*n* = 267)	MCI (*n* = 491)	AD (*n* = 119)	P value
Age, mean (SD), years	74.3 (5.9)	72.2 (7.5)	75.2 (8.1)	< 0.001
Female sex, No. (%)	131 (49.1)	212 (43.2)	49 (41.2)	0.211
Education, mean (SD), years	16.3 (2.7)	16.1 (2.7)	15.2 (2.9)	0.001
MMSE score, mean (SD)	29.0 (1.1)	27.7 (1.7)	23.2 (1.8)	< 0.001
APOE-e4 positive, No. (%)	73 (27.3)	235 (47.9)	83 (69.7)	< 0.001
CSF Aβ1–42, mean (SD), pg/mL	1217.5 (471.5)	983.8 (459.6)	654.4 (318.0)	< 0.001
CSF tau, mean (SD), pg/mL	242.4 (88.6)	287.6 (123.6)	364.8 (128.6)	< 0.001
CSF p-tau, mean (SD), pg/mL	22.3 (9.0)	27.8 (13.8)	36.5 (14.6)	< 0.001

Abbreviations: CN, control normal; MCI, mild cognitive impairment; AD, Alzheimer’s disease; MMSE, Mini-Mental State Exam; APOE, apolipoprotein E; CSF, cerebrospinal fluid; SD, standard deviation

**Table 2:** Serum bile acids and abbreviations

Abbreviation	Bile acid
12-KETOLCA	12-Ketolithocholic acid /12-Ketodeoxycholic acid
3-DHCA	3-Dehydrocholic Acid/ 3-Oxocholic Acid
7-KETOLCA	Nutriacholic acid / 7-Ketolithocholic Acid
7-DHCA	7-Dehydrocholic Acid
ALLOLCA	Allolithocholic acid (Isoallolithocholic acid)
APOCA	Apocholic Acid
CA	Cholic Acid
CDCA	Chenodeoxycholic Acid
DCA	Deoxycholic Acid
DEHYDROLCA	Dehydrolithocholic Acid
GCA	Glycocholic Acid
GCDCA	Glycochenodeoxycholic Acid
GDCA	Glycodeoxycholic Acid
GHCA	Glycohyocholic Acid
GHDCA	Glycohyodeoxycholic Acid
GLCA	Glycolithocholic Acid
GUDCA	Glycoursodeoxycholic Acid
HCA	Muricholic Acid\Hyocholic Acid
HDCA	Hyodeoxycholic Acid
ISOLCA	Isolithocholic acid
LCA	Lithocholic Acid
LCA_3S	Lithocholic Acid 3-Sulfate
MUROCA	Murocholic Acid
NORCA	Norcholic acid
NORDCA	3,12-Dihydroxynorcholanate\23-Nordeoxycholic acid
TCA	Taurocholic Acid
TCDCA	Taurochenodeoxycholic acid
TDCA	Taurodeoxycholic Acid
TUDCA	Tauroursodeoxycholic Acid
TAMCA	Tauro–muricholic Acid
UCA	Ursocholic Acid
UDCA	Ursodeoxycholic Acid
BUDCA	3-Ursodeoxycholic Acid (Isoursodeoxycholic acid)

### CSF biomarkers and serum BA

**Fig. 1:**
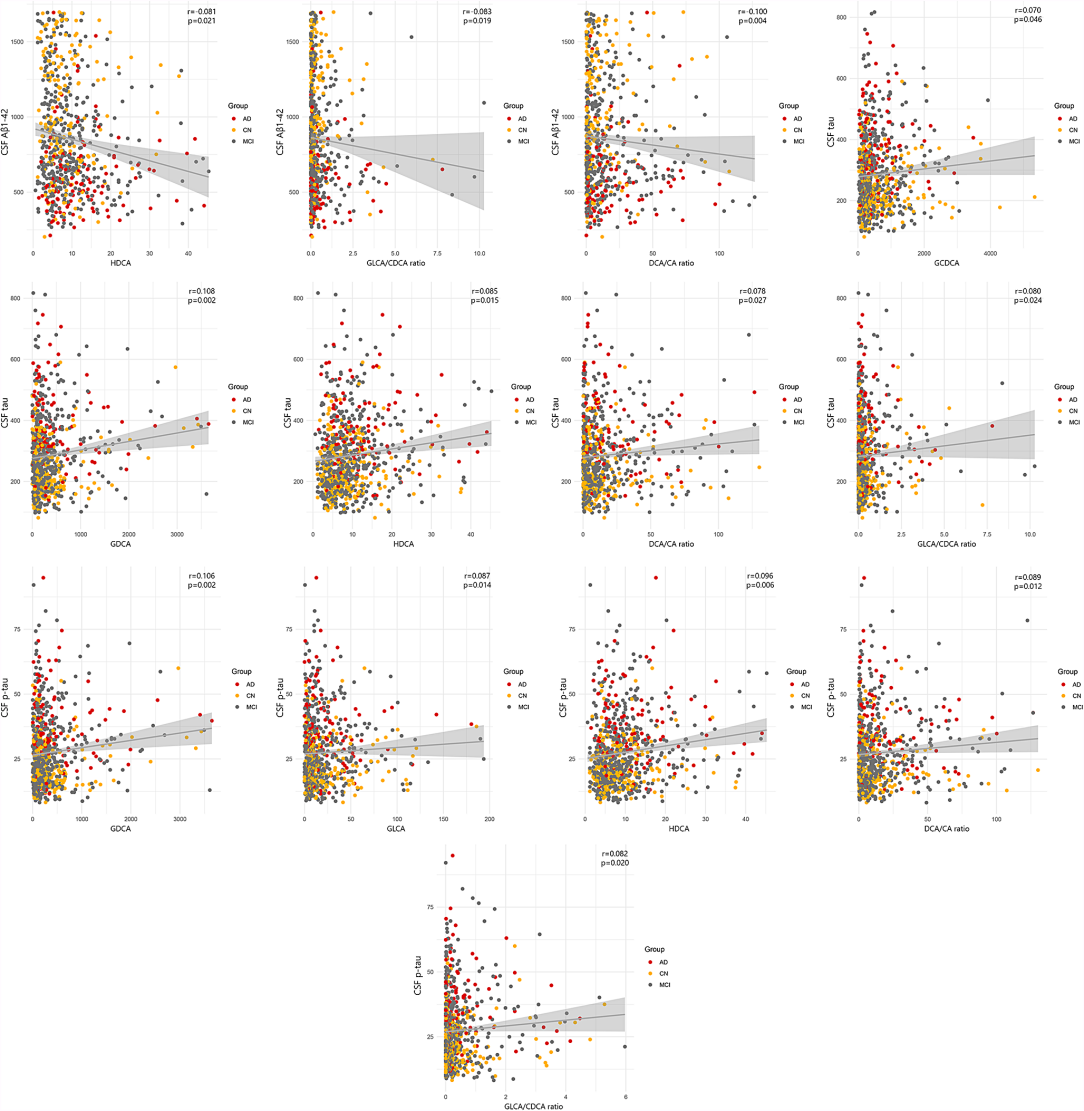
Significant associations between CSF biomarkers and BA profile after FDR correction

We investigated the association between cross-sectional CSF Aβ1–42, tau, and p-tau with 33 serum BA levels and 4 relevant ratios using multivariable linear regression models while adjusting for age, sex, APOE ε4, and BMI. After FDR correction we observed a significant correlation between CSF Aβ1–42 and one BA serum level and two ratios. The CSF Aβ1–42 was negatively associated with HDCA (r:-0.081, p:0.021), GLCA/CDCA ratio (r:-0.083, p:0.019), and DCA/CA ratio (r:-0.100, p:0.004) (Fig. 1).

Using the same model, we identified three BA serum levels and two ratios to be associated with CSF tau level (Fig. 1). We found that individuals with a higher level of CSF tau have higher serum BA levels including GCDCA (r: 0.070, p:0.046), glycodeoxycholic acid (GDCA) (r: 0.108, p:0.002), and HDCA (r: 0.085, p:0.015). Furthermore, there was a positive correlation between CSF tau with DCA/CA (r: 0.078, p:0.027) and GLCA/CDCA ratios (r: 0.080, p:0.024).

Also, we found that GDCA (r:0.106, p:0.002), GLCA (r:0.087, p:0.014), and HDCA serum level (r:0.096, p:0.006) were associated with CSF p-tau level among our participants (Fig. 1). Moreover, two BA ratios including DCA/CA (r:0.089, p:0.012) and GLCA/CDCA (r:0.082, p:0.020) were found to be correlated with CSF p-tau.

### PET imaging and serum BA

**Fig. 2:**
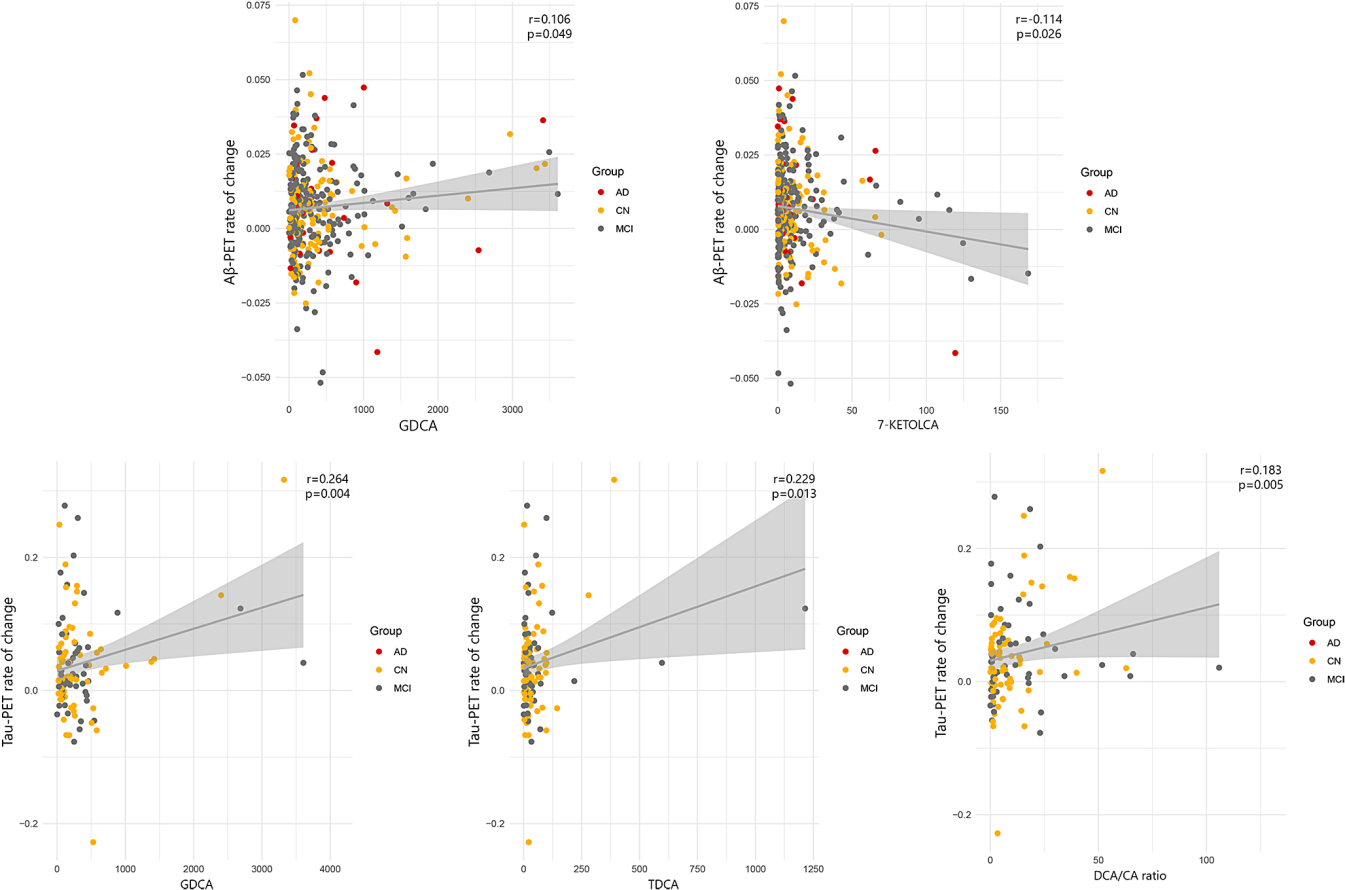
Significant associations between Aβ- and tau-PET and BA profile after FDR correction

We aimed to investigate whether the Aβ- or tau-PET is associated with the BA profile. Using the same regression model used for CSF biomarkers there was no significant association between baseline Aβ deposition and BA serum levels and ratios. However, when we examined the association between Aβ-PET rate of change and BA profile we found significant results (Fig. 2). The GDCA (r:0.106, p:0.049) and 7-KETOLCA (r:-0.114, p:0.026) were associated with the Aβ-PET rate of change.

Based on our analysis there was no significant association between baseline tau-PET and BA serum levels and ratios. While cross-sectional was non-significant, the longitudinal changes in tau-PET (rate of change) were associated with GDCA (r:0.264, p:0.004), taurodeoxycholic acid (TDCA) (r:0.229, p:0.013) level, and DCA/CA ratio (r:0.183, p:0.005) after FDR correction (Fig. 2).

## Discussion

In this study, we examined the BA profiles in the ADNI cohort to explore the correlation between peripheral metabolic indicators and central biomarkers related to AD pathophysiology. Additionally, we studied the connection between the rate of change in Aβ-PET and metabolic measures over time. The findings from our study indicated a significant relationship between BA profiles and three CSF biomarkers: Aβ1–42, tau, and p-tau. Our study revealed a negative association between levels of CSF Aβ1–42 and serum HDCA, as well as two ratios of secondary to primary BAs (DCA:CA and GLCA:CDCA). Additionally, a positive correlation was observed between CSF tau levels and serum GCDCA, GDCA, and HDCA levels, along with the same two secondary to primary BAs ratios. A positive correlation was also found between CSF p-tau levels and serum GLCA, GDCA, and HDCA levels, as well as the aforementioned ratios of secondary to primary BAs. Moreover, our longitudinal analysis revealed significant associations between the rate of change in Aβ-PET and levels of two BAs: GDCA and 7-KETOLCA. Additionally, we found that the rate of change in tau-PET was linked to levels of GDCA, TDCA, and the DCA:CA ratio.

Several studies investigated the association between serum BA concentrations and CSF and imaging biomarkers and symptoms of AD. Furthermore, a study on 1,464 subjects including cognitively normal, patients with MCI, and patients with AD showed that the while the serum levels of cholic acid (a primary BA) were significantly lower in AD patients compared to controls, AD patients had significantly higher serum levels of secondary bacterially-produced BAs including DCA and its glycine and taurine conjugated forms. Also, the ratios of secondary BAs to primary BAs such as DCA: CA, GDCA: CA, and TDCA: CA ratios were significantly associated with worse cognitive functions and were significantly higher in AD compared to MCI [9]. In another study, Nho et al. investigated the association between serum levels of 23 BAs and CSF and PET biomarkers of AD. They showed that GDCA: CA, TDCA: CA, and GLCA:CDCA ratios negatively correlated with CSF Aβ1–42 while there was a positive correlation between GDCA: CA ratio and amyloid deposition. Moreover, they found that higher secondary to primary BA ratios were significantly associated with higher CSF p-tau, lower hippocampal volume, and reduced brain glucose metabolism [10]. Additionally, in a longitudinal study, Varma et al. reported sex-specific associations between serum BAs and rates of brain atrophy indicating that in males, lower serum CDCA and CA levels were associated with faster rates of atrophy in total gray matter volume and various brain regions. However, in females, the results were controversial showing that in the Baltimore Longitudinal Study of Aging (BLSA) population, lower serum CA levels were associated with slower rates of total gray matter atrophy whereas in the ADNI population, lower CA levels correlated with faster rates of atrophy in only the fusiform gyrus and temporal gray matter [11].

The involvement of hepatic cholesterol metabolism is proposed to play a role in AD pathogenesis [12]. Cholesterol in the liver undergoes a process of synthesis to produce primary BAs, namely CDCA and cholic acid CA. These primary BAs are conjugated with glycine or taurine, then released into the gallbladder and subsequently transported to the intestine [13]. The liver-derived BAs are deconjugated and are further transformed into secondary BAs by intestinal anaerobic bacteria. For instance, CA is converted into DCA, while CDCA can be transformed into LCA or UDCA through either 7α-dehydroxylation or 7β-dehydroxylation, respectively [13–16]. Thus, The ratios of specific secondary to primary BAs, such as DCA:CA and GLCA:CDCA, typically indicate alterations in the gut microbial metabolism [16]. BAs are reabsorbed in the colon and terminal ileum and then released into the portal vein to be transported back to the liver, where they undergo conjugation to form their glycine and taurine derivatives [9].

Several metabolomics investigations conducted on individuals with AD or animal models have revealed substantial disparities in the composition and levels of BAs present in AD patients’ serum and brain tissue compared to healthy individuals [17–23]. A recent large-scale clinical study revealed that lower serum CA and CDCA concentrations were associated with faster brain atrophy in patients [11]. Moreover, the administration of supplemental UDCA, CDCA, and TUDCA has been shown to have beneficial effects in alleviating Aβ deposition, inhibiting Aβ-induced synaptic toxicity, reducing neuroinflammation, improving mitochondrial function, and ameliorating cognitive deterioration [24–28]. These findings indicate that altered BA profiles could contribute to cognitive dysfunction in AD and could potentially serve as a biomarker for early AD detection.

Interestingly, our study, for the first time, demonstrated that GDCA and 7-KETOLCA levels were associated with the Aβ-PET rate of change, and GDCA and TDCA levels and DCA:CA ratio were associated with the tau-PET rate of change. The novel longitudinal patterns of association between specific BA profiles and PET biomarkers observed in our study indicate a potential mechanistic connection between peripheral and central biochemical changes, highlighting the potential role of the gut-liver-brain axis in AD pathophysiology. Recent research on the gut-brain axis has revealed an emerging consensus that BAs, as metabolites intricately linked to the intestinal flora, play a crucial role as messengers within this axis [29, 30].

Previous research suggests a link between gut microbiota and brain amyloidosis. Mouse AD models lacking intestinal microbiota exhibited remarkably lower cerebral Aβ pathology than control mice with normal gut microbiota populations. Introducing microbiota from control AD mice into germ-free AD models led to a significant increase in Aβ pathology [31]. A hypothesis posits that disrupted gut microbiota and increased intestinal permeability facilitate the accumulation of amyloids derived from bacteria in the gastrointestinal tract at both systemic and brain levels, consequently promoting the buildup of Aβ42, potentially contributing to hippocampal dysfunction [32–36]. In addition, differences in the bacterial taxonomic composition of fecal samples were observed between cognitively impaired patients and controls. These differences included decreased levels of Firmicutes, Bifidobacterium, and Eubacterium rectale and increased levels of Bacteroidetes, Escherichia, and Shigella in the microbiome of patients. Notably, these variations were correlated with CSF biomarkers in both the patient and the control groups [37, 38]. These findings provide additional support to the growing evidence linking the gut microbiota with the development of brain amyloidosis.

### Limitations

As an observational study, the ADNI and other similar cross-sectional investigations pose challenges in controlling for confounding variables and unraveling the cause-and-effect relationships. For instance, numerous factors, such as sleep patterns, medications, nutrition, and environmental influences, impact the gut microbiota population, potentially contributing to the onset of AD [39, 40]. In this research, we thoroughly investigated age, gender, APOE ε4, and BMI as possible confounding factors. However, additional factors, such as medication usage and sleep patterns, were not taken into consideration. Consequently, future experimental studies are required to unravel the intricate mechanisms through which BA influences AD progression and untangle the web of causality.

Given the lack of adequate data, we could not examine the longitudinal changes in BA profiles. While a connection has been uncovered linking altered gut microbiome, BA profiles, and AD, it’s important to note that correlation does not necessarily imply causation. The microbiome and BA metabolism might change due to AD-related factors such as weight loss, medication use, or sleep disruption [39, 41]. Up to now, there haven’t been any extensive longitudinal clinical studies that have gathered fecal samples to examine alterations in the composition of the gut microbiome thoroughly. To further elucidate the association between BA profiles, gut dysbiosis, and AD-related pathologies, future large-scale studies are required to investigate these relationships over an extended period of time.

## Conclusions

To our knowledge, this is the first study examining the link between altered BA profiles and the longitudinal shifts in brain biomarkers, as identified through PET imaging. The findings from our study suggest a correlation between altered profiles of secondary BAs and CSF biomarkers associated with AD. These results provide supporting evidence for the link between the gut microbiome and the pathological features of AD. Furthermore, through our longitudinal analysis, we revealed novel connections between the pace of alteration in Aβ-PET and tau-PET and the specific BA profiles, suggesting a possible mechanistic link between peripheral and central biochemical transformations and shedding light on the potential involvement of the gut-liver-brain axis in the pathophysiology of AD. Although our findings offer additional support for the involvement of BA signaling pathways in AD, the precise cause-and-effect relationship still demands comprehensive exploration through experimental studies. Moreover, future extensive longitudinal studies are necessary to establish the connection between altered BA profiles, gut microbiome, and AD-related pathology and to elucidate intricate mechanisms through which gut bacteria and BAs contribute to the pathophysiology of AD.

## Data Availability

The datasets analyzed during the current study are available upon request with no restriction.
